# Supplementary material for: The realization logic of rural revitalization: Coupled coordination analysis of development and governance
Source: PLoS One. 2024 Jun 25;19(6):e0305593. doi: 10.1371/journal.pone.0305593 (PMC11198816; doi:10.1371/journal.pone.0305593)
Supplement: Attachment — Submitted filename: Response to Reviewers.docx [file pone.0305593.s002.docx]

**Response to Reviewers 1**

**[Comments 1]** The abstract is too lengthy. Please briefly describe the background, clarify the scientific problem, and the results need to be concise and concise. The conclusion needs to be further summarized and condensed.

**Response:** Thank you for your valuable comments to us. We rewrote the abstract, especially the background(Pg1, Ln12-33), methods(Pg1, Ln35-45), and results(Pg2, Ln47-68), to make it more precise. Specific changes can be found in a marked copy of the manuscript.

**[Comments 2]** L32-34, please check the language gramma of the two sentences.

**Response:** Thank you for your valuable comments to us. We rewrote the entire paragraph, using shorter sentences instead of longer ones to make it more straightforward(Pg4, Ln143-153). Specific changes can be found in a marked copy of the manuscript.

**[Comments 3 & 4 & 6]** L130, please modify punctuation marks. & L132, please check the sentence. & Literature review: the author is only listing the research of previous scholars. Please highlight the main idea of each paragraph and enhance the logical coherence in order to better introduce the scientific issues of the article.

**Response:** Thank you for your valuable comments to us. We have put together responses to these questions because we have rewritten the content of the literature review. Compared with the previous literature review, we made the following improvements. First, we changed the literature review format to make each paragraph's main idea more prominent. For example, we divided research on rural development into four categories and research on rural governance into three categories and summarized each. Secondly, we reorganized the literature context according to the logic from shallow to deep, making the logic of the article review more coherent. Third, we summarize the shortcomings of past research and future development trends to lay a theoretical foundation for the study. Specific changes can be found in a marked copy of the manuscript(Pg5, Ln189-308).

**[Comments 5 & 8]** The language of the entire article needs further polishing and improvement.

**Response:** Thank you for your valuable comments to us. We have re-examined the language issues of the full text and made improvements mainly in the following aspects. The first is to change long sentences into short sentences, such as P4 and Ln148-153. Secondly, we modify ten first-person problems, such as P4 and Ln162-168. Finally, we specifically checked the use of articles "the," "a," and "an" and corrected 14 grammatical errors. We have also revised some other grammatical issues. Specific changes can be found in a marked copy of the manuscript.

**[Comments 7]** Materials and methods: consider merging the description of this section into the preface and adding descriptions of indicators in Tables 1 and 2, such as calculation formulas and measurement of qualitative indicators.

**Response:** Thank you for your valuable comments to us. We have added a description of the problem in the introduction. The description of the research framework in the Materials and Methods section is used to respond to the questions raised in the introduction. Still, the content of the description has been modified. The issues already discussed in the preface will not be discussed here. In addition, descriptions of some general problems have been deleted from this section and added to the preface. The revised article has a more reasonable structure(P8, Ln339-399). Regarding the indicator description, due to the impact of page length, it is not easy to add content to Tables 1 and 2. Therefore, we put the description and calculation process of the indicators into the main text(P12, Ln478-507).

**[Comments 9]** L310-311, Is this a reference to previous research? Please add.

**Response:** Thank you for your valuable comments to us. This result references previous results, and we have added a piece of literature here. This document is the result of our previous research and is used to support the conclusions in the article. [Heng X, Xiang HX. Consistency or Conflict: Policy Reason and Value Choice in Urban and Rural Community Governance. Administrative Tribune. 2023 Jan;30(01):118-125.]

**[Comments 10]** Data Sources: this section should be placed in “Materials and methods”.

**Response:** Thank you for your valuable comments to us. We have restructured and removed data sources from the results and added them to Materials and Methods(P14, Ln521-540).

**[Comments 11]** Discussion: the discussion is too lengthy and lacks comparison with others, requiring further improvement.

**Response:** Thank you for your valuable comments to us. We have re-adjusted the content of the discussion section and mainly made the following changes. First, the content of the discussion section has been streamlined to make it more focused. Secondly, a comparative analysis with related research results has been added to the discussion section, and valuable parts of other results have been borrowed. Third, we modified the structure of the discussion section to make it easier to read. Specific changes can be found in a marked copy of the manuscript(P17, Ln647-1048).

**[Comments 12]** Suggest placing the limitations of the conclusion section on this article in the discussion section, and highlight the innovative results of this study in the conclusion.

**Response:** Thank you for your valuable comments to us. We have removed the discussion of limitations in the Conclusion and added it to the main body of the Discussion. In addition, we modified the conclusion's content to highlight this study's innovative results.

**Response to Reviewers 2**

**[Comments 1]** Minimize the use of interrogative sentence structures in the introduction and prefer declarative sentences. Enrich the explanation of the research background, and emphasize the theoretical and practical significance of this study throughout the article.

**Response:** Thank you for your valuable comments to us. We deleted the introduction's interrogative expression and changed it to a declarative sentence. In addition, we rewrote parts of the introduction and added a description of theoretical and practical implications(P5, Ln187-202).

**[Comments 2]** Provide specific points and descriptions in the conclusion and discussion sections.

**Response:** Thank you for your valuable comments to us. We have rewritten the Conclusion and Discussion sections. In the discussion section, we have made the following optimizations: First, we have streamlined the content to make it more specific. Secondly, a comparative analysis with related research results is added to the discussion section to highlight the uniqueness of this study. Third, we modified the structure of the discussion section to make it easier to read. In the Conclusion section, we have removed the discussion of limitations and added it to the main body of the Discussion. In addition, we modified the conclusion's content to highlight this study's innovative results(P17, Ln647-1108).

**[Comments 3]** The literature review should not merely list authors and their works but should elaborate on their viewpoints and significant contributions to the field. Address shortcomings in past studies and highlight the innovations and advantages of the current research in comparison.

**Response:** Thank you for your valuable comments to us. Compared with the previous literature review, we made the following improvements. First, we changed the literature review format to make each paragraph's main idea more prominent. For example, we divided research on rural development into four categories and research on rural governance into three categories and summarized each. Secondly, we reorganized the literature context according to the logic from shallow to deep, making the logic of the article review more coherent. Third, we summarize the shortcomings of past research and future development trends to lay a theoretical foundation for the study. Specific changes can be found in a marked copy of the manuscript(Pg5, Ln189-308).
