# Supplementary material for: The realization logic of rural revitalization: Coupled coordination analysis of development and governance
Source: PLoS One. 2024 Jun 25;19(6):e0305593. doi: 10.1371/journal.pone.0305593 (PMC11198816; doi:10.1371/journal.pone.0305593)
Supplement: Attachment — Submitted filename: Response to Reviewers 2.docx [file pone.0305593.s003.docx]

**Response to Reviewers 1**

We greatly appreciate your help in further improving our research. Your valuable suggestions allow us to improve our research results further. Below are responses to your comments. Thank you again for your help.

**[Comments 1]** Firstly, the language of the whole text needs further refinement.

**Response:** Thank you for your valuable comments. We checked the language issues of the full text through self-checking and help from others. We have modified the language expression of all contents, including abstract, main text, acknowledgments, etc. Pay special attention to issues such as articles, tenses, and persons. At the same time, we modified the long sentences of the article to express its meaning better. Finally, we reconfirmed the expression of some proper nouns to ensure their correctness. We have retained traces of modifications in the manuscript for your review.

**[Comments 2]** Secondly, the conclusion section, especially the recommendations, in the abstract should be specific. For example, what are the three coupling coordination paths?

**Response:** Thank you for your valuable comments. There is indeed a problem with our previous expression. We have made corrections to this version. We have added detailed research conclusions, countermeasures, and suggestions to make the abstract more specific (Pg2, Ln47-54).

Thank you again for your help with our research.
